# Supplementary material for: A rapid calprotectin test for the diagnosis of pleural effusion
Source: PLoS One. 2021 Jun 10;16(6):e0252714. doi: 10.1371/journal.pone.0252714 (PMC8191907; doi:10.1371/journal.pone.0252714)
Supplement: S1 Table — (DOCX) [file pone.0252714.s001.docx]

**S1 Table.** Diagnostic accuracy of the pleural fluid calprotectin levels measured by ELISA fCAL^®^.

| Cut-off^*^ | S (%) | E (%) | PPV (%) | NPV (%) | LR^+^ | LR^–^ | AUC |
| --- | --- | --- | --- | --- | --- | --- | --- |
| *≤* 6,233.2 | 96.9  (91.3–98.3) | 60  (53.6–66-1) | 57.6  (51–64) | 96.5  (92.1–98.5) | 2.40  (2.04–2.83) | 0.06  (0.03–0.15) | 0.848  (0.810–0.886) |

Data are presented as percentages and 95% confidence intervals. ^*^Calprotectin levels are expressed in ng/mL.

S = sensitivity; E = specificity; PPV = positive predictive value, NPV = negative predictive value, LR^+^ = positive likelihood ratio, LR^–^ = negative likelihood ratio, AUC = area under the curve.
