# Supplementary material for: A rapid calprotectin test for the diagnosis of pleural effusion
Source: PLoS One. 2021 Jun 10;16(6):e0252714. doi: 10.1371/journal.pone.0252714 (PMC8191907; doi:10.1371/journal.pone.0252714)
Supplement: Attachment — Submitted filename: 210407 Response to Reviewers..docx [file pone.0252714.s005.docx]

Lorena Vázquez Iglesias

CINBIO. Universidad de Vigo.

36310 Vigo. Spain.

Email: [lorena.vazquez@uvigo.es](mailto:lorena.vazquez@uvigo.es)

Pal Bela Szecsi, M.D. D.M.Sci.

Academic Editor of *PLOS One*

#### Vigo, 7^th^ April 2021

Dear Editor,

We would like to thank you and the reviewers for the careful evaluation and for your invitation to submit a revised version of our work **“A rapid calprotectin test for the diagnosis of pleural effusion”**.

In an attached rebuttal letter we provide point-by-point responses to all of the issues raised by the reviewer, indicating the extent to which appropriate changes have been implemented in our revised text.

As required, we are also sending a marked-up version of the manuscript that shows the changes made to the original article highlighted in red and a clean revised version (unmarked version) of the manuscript.

Sincerely,

Lorena Vázquez Iglesias

**ANSWERS TO PLOS One Editor**

Changes have been highlighted in red in a new version of the manuscript for an easier revision.

**Comment 1: In adding to the issues raised by the reviewers, please clarify the main purpose of the paper. Is a validation of using calprotectin in pleural fluid as a surrogate for leucocytes, in diagnosing malignant or infections effusions? Or is it a comparison between calprotectin POCT and ELISA methods.**

**Response 1:** We apologize for the confusion regarding the main purpose of this manuscript. According to your request, we correct the objective to clarify the main purpose of the paper**.** We intend to mean that calprotectin is a complementary biomarker to the rest of the biochemistry parameters and that could help in the differential diagnosis of PE when we perform an urgent thoracentesis. At this time, the gold standard technique used for evaluation of calprotectin is the ELISA method, but this method cannot be used in urgent situations. We correct the main objective in the manuscript as follows: *The objective of this study was to evaluate a rapid method to measure calprotectin levels in PF samples in urgent situations for differentiating between benign (BPE) and malignant pleural effusion (MPE) and compare it with the ELISA method.*

**Comment 2: Please explain abbreviations at first occurrence if used > 3times, remember the abstract is a separate unity.**

**Response 2**: We apologize for this mistake. Following this advice, we correct this in the manuscript.

**Comment 3. The manuscript could benefit from native English editing**

**Response 3.** As suggested by the Editor, the manuscript has been reviewed by a Native English Editors team.

**ANSWERS TO Reviewer 1**

We would like to thank the comments about our work. We have considered the questions and comments raised by the referee and here we provide the itemized answers. Changes have been highlighted in red in a new version of the manuscript for an easier revision.

**Comment 1:** **In the pag 4 - Methods - the authors report that only "healthy adults over 18 years were eligible for the study..." If they have pleural effusion, they are not healthy!! I suggest correction**.

**Response 1:** We apologize for the mistake and we corrected this sentence in the manuscript: *Only adults over 18 years were eligible for this study.*

**Comment 2: In the page 10 - Results - table 3: The values of Calprotectin presented as 5000 ng/mL in MPE, does it mean that there was no detection with the assay used? If the lower detection value was considered for cases with Calprotectin undetectable by the assay used, this criterion did not prejudice the statistical analysis?**

**Comment 2.1: was no detection with the assay used?**

**Response 2.1**: As we commented in methods section (Assay validation)**,** the measurement range of QB^®^sCAL is 5000 to 100000 ng/mL. This does not mean that lowers values are no detected. In this test, it is recommended to assign the values of the detection limits (upper or lower) to those samples that present values above or below the detection limits.

**Comment 2.2: If the lower detection value was considered for cases with Calprotectin undetectable by the assay used, this criterion did not prejudice the statistical analysis?**

**Response 2.2:** We are in agreement with the referee that this criterion could be affect the statistical analysis. In the data sheet of Quantum Blue® sCAL assay says: The results showed linearity within the indicated measuring range of 5000to 10000 ng/mL of the Quantum Blue® sCAL assay. For that reason, we decide to apply this criterion to do the analysis even though that the statistical analysis could be worse.

**Comment 3: The authors should refer to the average cost of the test and the equipment, especially considering its use in developing countries**;

**Response 3***:* According to your request the average cost of calprotectin measurement by the Quantum Blue method has a cost of 20 euros per test. This price includes the cost of reagents and the loan of the equipment (Quantum Blue Reader) to the clinical laboratory by Bühlmann company. Initially, we have not included this data in the manuscript because we think that it does not contribute to the research, but if you consider that it is important we include it in the manuscript.

**Comment 4:** **In the study there is no reference to the positivity of the PE cytology. What was the criterion used in the definition of MPE: cytology or biopsy? It would be interesting to report on the performance of cytology in the case of MPE.**

**Response 4:** As suggested by the referee we add this sentence in methods section:  *The MPE was diagnosed by cytological or histologic examination*. *Malignant origin was defined when malignant cells were identified upon cytological or histological examination or in a biopsy specimen.*

In the discussion section we add this comment: *Pleural fluid cytology has traditionally been the analytical method of choice for the detection of tumor cells in pleural fluid. However, pleural fluid cytology is far from perfect for diagnosing MPE. First, the diagnostic sensitivity of pleural fluid cytology varies and is higher (up to 60%) in metastatic malignancies than in mesothelioma (~30%). Second, sensitivity is at least in part dependent on the experience of the cytologist, the tumor load and the amount of fluid submitted. Another limitation is that standard cytology examination based on cytomorphology is often unable to distinguish between different types of malignancy (e.g., between adenocarcinoma and mesothelioma) without the use of special studies such as immunohistochemistry. Immunohistochemistry complements cytology in the diagnosis of malignant pleural effusions. But the patient’s cytology in conjunction with immunohistochemistry. studies will not establish a diagnosis, and additional investigations will be required – usually invasive procedures to sample pleura for histological examination.*

**Comment 5**: **Considering the values obtained for specificity, PPV and diagnostic accuracy, would the authors really suggest using this POCT assay for clinical management??**

**Response 5:** According to your request*,* we considering that this POCT assay could be uses for clinical management. The principal reason is that our study confirms that calprotectin levels measured with this POCT assay in combination with clinical variables, has an excellent discriminatory value for predicting suspected MPE in medical emergency. The QB^®^sCAL achieves good accuracy in PE calprotectin measurement with a good discrimination between MPE and BPE patients. For a proportion of patients with pleural effusions, pleural fluid cytology not establish a diagnosis, and additional investigations will be required, usually invasive procedures to sample pleura for histological examination. QB^®^sCAL is a noninvasive diagnostic tool that should be implemented in clinical practice.

**ANSWERS TO Reviewer 2**

We would like to thank the comments about our work. We have considered the questions and comments raised by the referee and here we provide the itemized answers. Changes have been highlighted in red in a new version of the manuscript for an easier revision

**Comment 1: Some abbreviations in this paper are not given full names, so that the paper is less readable**.

**Response 1:** We apologize for the mistake of not referring the full names of some abbreviations. We have been corrected the errors.

**Comment 2**: **What is the main contribution of this paper? Is the calprotectin level measurement method proposed in this paper for the first time? If so, this paper should focus on the proposed measurement method. Or does the experiment prove that the method can be used in clinical practice? The contribution of this article should be clearly written**.

**Response 2***:* We apologize for the confusion regarding the main purpose of this manuscript. Both arguments are right. This is the first time that this calprotectin measurement method (QB^®^sCAL) is used in PF samples and also this study confirms that this method can be used in clinical practice. According to your request, we correct the objective to clarify the main purpose of the paper**.** We intend to mean that calprotectin is a complementary biomarker to the rest of the biochemistry parameters and that could help in the differential diagnosis of PE when we perform an urgent thoracentesis. At this time, the gold standard technique used for evaluation of calprotectin is the ELISA method, but this method cannot be used in urgent situations. We correct the main objective in the manuscript as follows: *The objective of this study was to evaluate a rapid method to measure calprotectin levels in PF samples in urgent situations for differentiating between benign (BPE) and malignant pleural effusion (MPE) and compare it with the ELISA method.*

Furthermore, the text in Discussion section has also been modified to explain the this better, as follows: *Our study confirms that calprotectin levels measured with a rapid quantitative lateral flow assay in combination with clinical variables, has an excellent discriminatory value for predicting suspected MPE in medical emergency.*

*In conclusion, a rapid quantification of PF calprotectin using QB^®^sCAL assay represents a fast and reliable method for diagnosing of MPE and it could be an alternative to the ELISA method. The QB^®^sCAL achieves good accuracy in PE calprotectin measurement with a good discrimination between MPE and BPE patients. QB^®^sCAL is a non-invasive diagnostic tool that should be implemented in clinical practice.*

**Comment 3:** **The advantages of the Quantum Blue® sCAL measurement method are mentioned in the paper, but the disadvantages of this method are not clearly pointed out, which should be supplemented in this paper**.

**Response 3:** As suggested by the referee, we have added the disadvantages of the method. We added this part in the manuscript: *On the other hand, it is important to mention that a principal limitation of this method is the preanalytical treatment of PF samples because the samples require a centrifugation and dilution prior the analysis. This preanalytical treatment require adequate laboratory equipment that could limit the use of the Quantum Blue® sCAL method outside the emergency laboratories.*

**Comment 4:** **In the assay validation part, is it reasonable to take samples of 5000-24000 to analyze the correlation between the two measurement methods? Need to point out the reason for this analysis, or supplement the correlation analysis of the two measurement methods on 253 samples**.

**Response 4:** As stated in the methodology section (Assay validation)**,** due to the difference in the linearity range of both tests following optimization for use in PF, the analytical comparison of this methods was only possible made with the 48 samples that were included in the measurement range shared by both assays (5000-24000 ng/mL). However, the clinical concordance is independent of this linearity range of the methods used because of that all the samples (253) were included. As we explained, we calculated a cut-off point for each method and categorized the samples as positive and negative based on that cut-off point.

**Comment 5**. **The paper does not give the diagnosis result of calprotectin level measured by ELISA method, and the result similar to Table 4 for ELISA method should be given.**

**Response 5***:* We agree with your comment and we add the diagnostic accuracy of pleural fluid calprotectin for ELISA method*.*

**Comment 6:** **Does the cut-off value in this paper have guiding significance for the follow-up clinical diagnosis of pleural effusion?**

**Response 6:**

This is a very interesting comment and it may be possible that the calprotectin values could be used in the follow-up of the pleural effusion diagnosis but unfortunately the calprotectin measure for this study only was made in the initial time.

**Comment 7**: **there are many relevant predictive models have been proposed for the diagnosis of pleural effusion, the authors should make a deep investigation on this issue in the introduction part, such as the works [1]- [4].**

**Response 7:** According to your request, we revised these references and redacted the introduction section including them.
